# The effect of yoga practice on glycemic control and other health parameters in the prediabetic state: A systematic review and meta-analysis

**DOI:** 10.1371/journal.pone.0221067

**Published:** 2019-10-16

**Authors:** Ramya Ramamoorthi, Daniel Gahreman, Timothy Skinner, Simon Moss

**Affiliations:** 1 College of Health and Human Sciences, Charles Darwin University, Darwin, Northern Territory, Australia; 2 Københavns Universitet, Institut for Psykologi, Center for Sundhed og Samfund, Københavns Universitet, København K, Denmark; Tabriz University of Medical Sciences, ISLAMIC REPUBLIC OF IRAN

## Abstract

A systematic review and meta-analysis was conducted to investigate the effects of yoga on glycemic control, lipid profiles, body composition and blood pressure in people in the pre-diabetic state. Studies on the effectiveness of yoga on population groups under high risk for diabetes, called prediabetic or suffering from metabolic syndromes were extracted from a thorough search of PubMed, Scopus, Cochrane Library, EBSCO and IndMED databases. Both Randomised Controlled Trial (RCT) and non-RCT studies were included in the systematic review and meta-analysis. Studies published between Jan 2002 and Dec 2018 were included. Studies were considered for evaluation if they investigated a yoga intervention to prevent T2DM, against a control group, while also reporting glycemic control and other health parameters of T2DM management. Summary effect sizes and 95% confidence intervals (CI) were calculated using the Comprehensive Meta-Analysis software in addition to publication bias. Of the 46,500 identified studies, 14 studies with 834 participants of whom were 50% women, were found to be eligible for inclusion in our systematic review. Our quantitative synthesis included 12 randomized control trials and 2 non-randomized control trials, with the follow-up period ranging from 4 to 52 weeks. Compared to controls, yoga intervention improved fasting blood glucose (FBG) [Standard Mean Difference (SMD -0.064 mg/dL (95% CI -0.201 to 0.074)]; low density lipoprotein (LDL) [SMD-0.090 mg/dL (95% CI -0.270 to 0.090)]; triglycerides [SMD -0.148 mg/dL (95% CI -0.285 to -0.012)]; total cholesterol [SMD -0.058 mg/dL (95% CI -0.220 to 0.104)] and systolic blood pressure [SMD -0.058 mm Hg (95% CI -0.168 to 0.053)]. This meta-analysis uncovered clinically improved effects of yoga intervention on glycemic control, lipid profiles and other parameters of T2DM management in prediabetic population. These results suggest that yoga intervention may be considered as a comprehensive and alternative approach to preventing T2DM. Further adequately powered, well designed RCTs are needed to support our findings and investigate the long-term effects of yoga in T2DM patients.

## Introduction

Type II diabetes mellitus is a chronic metabolic disease characterized by persistent hyperglycemia because of a progressive condition in which the body becomes resistant to the typical effects of insulin or loses the ability to produce insulin [1]. The global prevalence of diabetes among adults above 18 years old has increased to 8.5% in 2014 from 4.7% in 1980. Approximately 422 million people were found to be living with diabetes in 2014 [2] and approximately 1.6 million deaths were directly caused by diabetes in 2016 [3].

The key risk factors often associated with the development and maintenance of Type 2 diabetes mellitus (T2DM) include sedentary lifestyle [4], or an unhealthy diet and psychological stress. Psychological stress is strongly associated with both the risk factors [5–7] and maintenance of the disease [8, 9]. In addition to the genetic background, the prediabetic state also contributes significantly to the development of T2DM [4, 10].

The critical components of diabetes management are medication, diet and physical activity/exercise [11]. However, many complementary and alternative practices have been used by people in both the prevention and treatment of diabetes [12, 13] such as yoga. Yoga originated in India over 5000 years ago as a form of traditional mind-body training [14, 15]. The efficacy of yoga has been studied in several chronic diseases, such as hypertension, asthma, chronic obstructive pulmonary disease and diabetes [16–18].

Previous studies have reported that the practice of yoga might reduce Insulin Resistance Syndrome, is an exclusive collection of risk factors for the development of T2DM and have shown promising results in improving signs, improving prognosis and reducing complications [16, 18–22]. Furthermore, studies showed the development of diabetes from the prediabetic state could be either delayed or ameliorated by consistent physical activity [23–26], healthy diet [24] and active stress management [27, 28].

Previous studies have highlighted that yoga could lessen fasting blood glucose (FBG) and glycosylated hemoglobin A1c (HbA1c) as well as reduce the lipid levels while improving the quality of life of T2DM patients [29–36]. However, these studies showed inconclusive results with wide variations in their sample size. A limitation of the study may include the inclusion of a few studies based on a non-randomized study design, not informed so could potentially impact the outcome [32, 33, 35]. Nevertheless, a few previously published systematic reviews have reported the efficacy of yoga on blood glucose levels, insulin sensitivity, oxidative stress, lipid profile, anthropometric measures, pulmonary measures, nerve conduction and quality of life for T2DM with promising results [37–40]. Raveendran and colleagues demonstrated the role of various yoga practices in the management of diabetes based on evidence from various clinical studies [41].

The promising benefits of yoga interventions for T2DM have also been recorded in a recent meta-analysis. Innes and Selfi demonstrated the impact of yoga among adults with T2DM to improve glycemic control, lipid levels and body composition measured by body weight and mass index from a systematic review of 25 controlled trials [42]. Cui and colleagues reported a meta-analysis of 12 randomized controlled trials and highlighted the progress in pooled weighted mean differences for fasting blood glucose and hemoglobin A1c. The authors of this particular study also measured the weighted effect size of yoga efficacy for postprandial blood glucose (PPBS), total cholesterol, high-density lipoprotein cholesterol, low-density lipoprotein cholesterol and triglycerides [11]. Kumar and colleagues documented beneficial effects of yoga as a complementary intervention to standard treatment in comparison to standard treatment on FBS for PPBS and HbA1C [43]. Thind and colleagues showed, in their meta-analysis that yoga T2DM participants were successful in improving their HbA1c, FBG and PPBG, in addition to significant improvements in lipid profile, blood pressure, body mass index, waist/hip ratio and cortisol level [44]. Because past meta-analysis has not been conducted on the effects yoga on prediabetic populations, we carried out a meta-analysis of RCT and non-RCT studies in the present study to determine the effectiveness of yoga in patients with high-risk T2DM. We hypothesize that yoga intervenes T2DM by two proposed mechanisms: downregulation of both the hypothalamic pituitary adrenal axis and the sympathetic nervous system [45–47].

### Rationale

#### What is the issue and how will our study address this?

Studies published previously in the prediabetic population showed the effectiveness of yoga in reducing the risk of progression to diabetic state [48–50]. There are a few systematic reviews and meta-analyses that demonstrate the efficacy of yoga in T2DM. However, there is currently no systematic review and meta-analysis study that details the potential benefits of yoga in the prediabetic population. This is the first such study to show evidence that yoga intervention may impact the prediabetic state by assessing the glycemic control (HbA1c, fasting blood glucose (FBG) and postprandial glucose (PPBG) in both the intervention and control group conditions. This study also summarises the results of other markers of diabetes management including triglycerides, high-density lipoprotein, low-density lipoprotein, systolic and diastolic blood pressure, body composition and fasting cortisol, as influenced by yoga in prediabetic populations. The study provides further evidence that yoga intervention could be considered as an effective alternate treatment or lifestyle therapy for people who are under high risk of T2DM.

### Review questions

Our systematic review and meta-analysis sought to clarify the association between yoga intervention and prediabetic state. We also compared the overall effect of the yoga intervention on glycemic control and other markers of diabetes management.

The questions for this review are as follows:

- Does yoga delay or prevent the progression of diabetes in a prediabetic population?
- What is the significance of yoga compared to exercise in a prediabetic population?
- How much does the effect size of physiological outcomes vary between studies?

## Methods

This systematic review and meta-analysis was registered in the PROSPERO (Registration number CRD 42018106657) database and followed the Preferred Reporting Items for Systematic Review and Meta-Analysis (PRISMA) guidelines [51]. This protocol has been published in Medicine Journal.

### Search strategy

**Fig 1:**
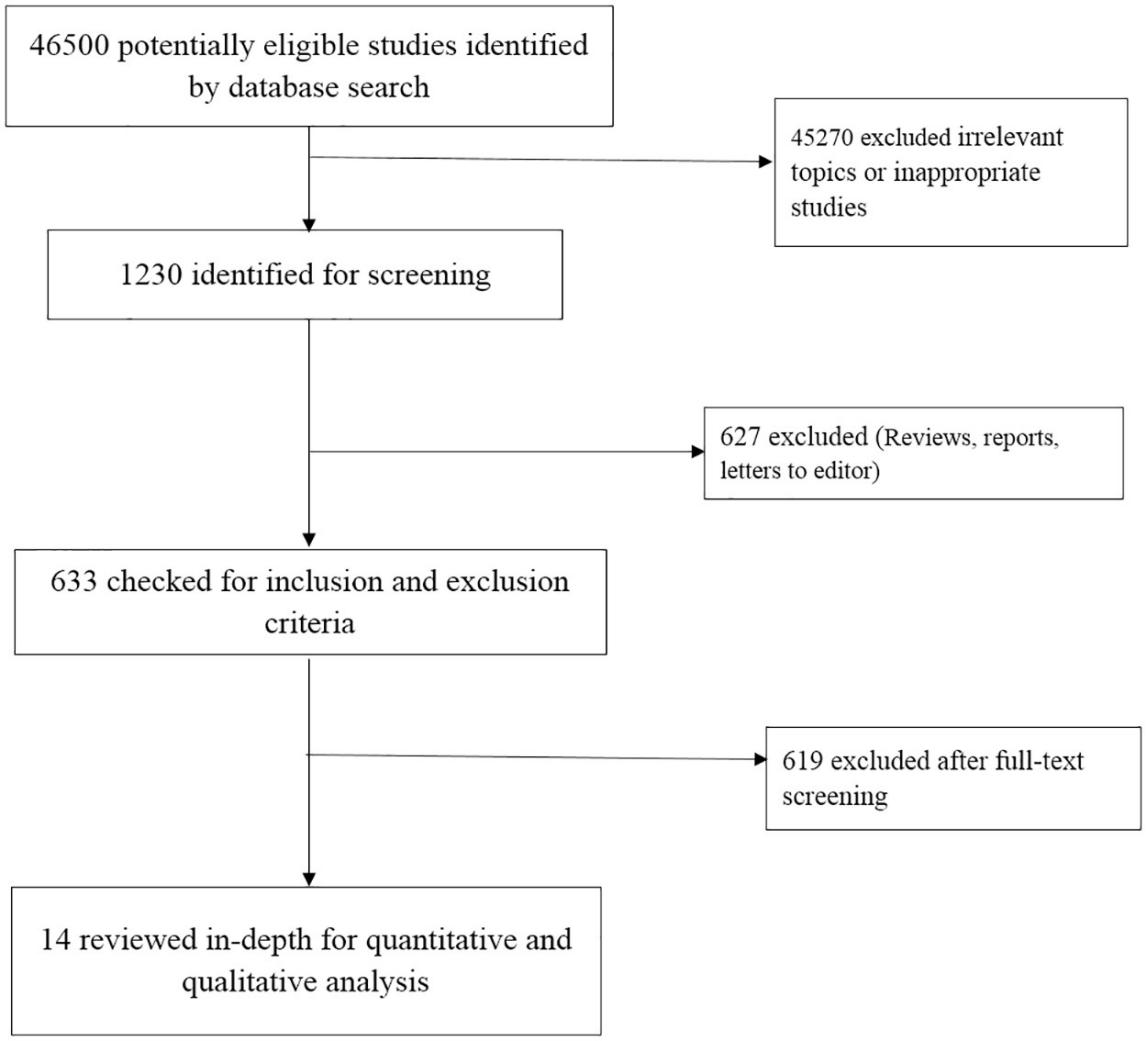
A flowchart depicting the study selection and the search results.

We searched 5 electronic bibliographic databases: PubMed, Scopus, Cochrane Library, EBSCO and IndMED using a combination of search terms for the effectiveness of yoga on people with high risk of diabetes or prediabetic population, or metabolic syndrome ((yoga, cardiovascular disease risk factors, prediabetes state, high risk for diabetes, metabolic syndrome, glucose, type II diabetes, exercise therapy and type 2 diabetes patients)) [52]. Two authors performed title and abstract screening and then accessed potential full-text eligible articles. The corresponding author performed a final assessment on eligible studies for inclusion, and the references of eligible studies were imported into an EndNote file to create an initial list of eligible studies and avoid duplication. Any discrepancies associated with the selection of the studies were resolved by mutual discussions involving a third reviewer. The entire selection process is illustrated in Fig 1.

### Study selection criteria

The included studies had to meet the following (PICO) criteria

#### Inclusion criteria

- Participants (P): Participants with prediabetic or designated as high risk for diabetes because of physiological measures.
- Intervention (I): Inclusion of studies was dependent on the program following authentic or traditional yoga rules and techniques. Studies examining yoga intervention including at least one of asana, pranayama, meditation to promote T2DM management and comparing it with a control. Studies with randomised control trial’, randomised cross-over studies, cluster-randomised trials, or quasi-experimental design were included.
- Control (C): Any control with other usual care or physical exercise or non-therapeutic intervention
- Outcomes (O): Studies evaluating glycemic control as well as other measures of T2DM management such as HbA1c, blood pressure, fasting blood sugar, lipid profile (triglycerides, high and low-density lipoprotein (HDL and LDL) cholesterol), systolic blood pressure and diastolic blood pressure in both the intervention and control group conditions.

#### Exclusion criteria

- We excluded study participants who were members of a specific age group, such as adolescents or geriatric age groups
- Study participants were all in a transient state, such as pregnancy or menopause
- Studies were excluded if the yoga intervention was modified to a dance program
- Studies from conference proceedings, editorials, commentaries, book chapters and book reviews were excluded.

### Data extraction and management

Two authors developed a data extraction form using MS Excel and independently evaluated the published studies with the predefined selection criteria for inclusion into the review. Data were extracted based on six categories: 1. Study information (first author, year of publication, country, a journal of publication, the study period); 2. Study design and methods (a type of research, details of randomised control trial, randomised cross-over studies and cluster-randomised trials or quasi-experimental design and the validity of confirmative diagnosis and method of data collection); 3. Study participants characteristics (condition, age, gender, race, sample size and sampling procedures); 4. Yoga intervention characteristics (Yoga type: asana, pranayama, meditation, components, frequency, duration); 5. Control intervention characteristics (Type: usual care or physical exercise or non-therapeutic intervention, frequency, duration); 6. Outcome measures (Primary and secondary outcome measures, assessment time points, blood pressure (systolic, diastolic); heart rate; respiratory rate; abdominal obesity (waist circumference, waist-hip ratio, index of central obesity); blood lipid levels (triglycerides, high and low-density lipoprotein (HDL and LDL) cholesterol); glycemic control (both the intervention and control group conditions, such as HbA1c, blood pressure, or fasting blood sugar).

### Study outcomes

#### Primary outcome

The primary outcome was the measurement of glycemic control (HbA1c) fasting blood glucose (FBG) and postprandial glucose (PPBG).

#### Secondary outcomes

The secondary outcomes were other markers of diabetes management including triglycerides, high-density lipoprotein, low-density lipoprotein, systolic and diastolic blood pressure, body composition and fasting cortisol.

### Assessment of risk of bias

Risk of bias of included studies was assessed using the Cochrane Risk of Bias Assessment tool. This tool assesses several items under seven categories such as random sequence generation, allocation concealment, blinding of participants and investigators, the blindness of outcome assessments, incomplete outcome data, selective outcome reporting, and other biases. Based on the assessment, the studies were evaluated as low, unclear, or high bias. The PRISMA checklist is given in S1 Table.

The Jadad scale was used to evaluate the quality of each study in which three domains in the scale cover randomization (0–2 points), blinding (0–2 points), and dropouts and withdrawals (0–1 point) [53]. A trial with a score of ≤ 2 indicates low quality whereas a score of ≥3 indicates high quality. Assessment of publication bias was performed using funnel plots generated by Comprehensive Meta-Analysis (CMA) 3.0 software.

### Data synthesis

#### Meta-analysis-assessment of overall effect size

Meta-analyses were performed using CMA, and the effectiveness of yoga interventions on the glycemic status of the prediabetic population was computed. We combined effect sizes across included studies and reported pooled effect estimate as standardised mean difference (SMD) with their 95% Confidence Intervals (CI). Random-effects model of the meta-analysis was followed, and forest plots were generated to visually assess the pooled effect size of the study’s findings.

The outcome measures in this systematic review and meta-analysis were continuous. Negative SMDs indicated better performances of yoga interventions on the glycemic status and other markers of the prediabetic population or the beneficial effects of yoga for diabetes risk [43].

### Assessment of heterogeneity

Heterogeneity in effect size was assessed using the Cochrane Q test and I^2^-statistic. Q statistics and the associated degree of freedom were estimated for each outcome. Q statistics provides a test of the null hypothesis that all studies in the proposed meta-analysis share a standard effect size. If all studies shared the same effect size, the expected value of Q would be equal to the degrees of freedom the number of studies minus 1. I^2^ statistics reflect the proportion of the observed variance that informs the difference in true effects sizes rather than the sampling error of the included studies. The tau-squared parameter indicates information of the heterogeneity between the effects for the test accuracy observed in different studies [54].

### Publication bias

Publication bias of the included studies was assessed using Egger’s bias indicator test. Orwin’s [55] and classic fail-safe N test’ [56], ‘Begg and Mazumdar Rank correlation test’ [57], and ‘Duval and Tweedie’s Trim and Fill ‘calculations [58] were used to impute missing small studies with large effect size to be dispersed equally on either side of the overall effect, to provide a more accurate estimate of the likely publication bias

#### Funnel plot

The funnel plot is a plot of study size (standard error or precision) on the vertical axis versus a function of effect size (standard mean difference) on the horizontal axis. The included studies in the plot are distributed symmetrically about the combined effect size in the absence of publication bias. This plot was generated using Egger’s test [59]. Conversely, a higher concentration of studies is observed on one side of the mean than the other in the presence of bias.

#### Classic fail-safe N and Orwin fail-safe N

The critical reason for publication bias is that some non-significant studies are missing from this meta-analysis. The observed effect of yoga intervention will be invalidated if these missing studies are included in the analysis. If the missing number is relatively small, publication bias is likely. However, if these missing number of studies are large, the findings are interpreted as the intervention effect of yoga could be possibly inflated by the exclusion of some studies, is nevertheless not nil [60]. Both Classic and the Orwin fail-safe N reports the likelihood that studies are absent from the analysis and that these studies if included in the analysis, would shift the effect size toward the null [55].

#### Begg and Mazumdar rank correlation test

In general, extensive studies tend to be included in the quantitative synthesis or meta-analysis regardless of their yoga intervention and control group effects while small studies are more possible to be included when they display a comparatively sizeable intervention effect. Therefore, an inverse correlation between study size and effect size will tend to be observed. Begg and Mazumdar proposed that this kind of correlation can aid as a test for publication bias [61]. Therefore, the rank order correlation was computed (Kendall's tau b) between the intervention effect and the standard error (which is driven primarily by sample size).

#### Egger's test of the intercept

The Egger regression yields the degree of funnel plot asymmetry or the same bias by using precision -the inverse of the standard error to predict the standardised effect (effect size divided by the standard error). The Egger's Test of the Intercept was undertaken to gauge the relationship between test accuracy estimates and their precision [59].

#### Duval and Tweedie's Trim and Fill

Duval and Tweedie's Trim and Fill test imputes the missing studies of both yoga and control conditions are, add them to the analysis, and then recomputes the combined intervention effect. The *'Trim and Fill'* method iteratively prunes the asymmetric studies from the right-hand side to localize the unbiased effect. Then this method completes by re-introducing the trimmed studies on the right as well as their imputed counterparts to the left of the mean intervention effect [62].

### Subgroup analyses

Subgroup analyses were performed based on study, participant and outcome characteristics and methodological factors. We have investigated specific subgroup analyses according to differences in intervention and critical features of identified study participants such as glycemic control (both the intervention and control group conditions, such as HbA1c, blood pressure, or fasting blood sugar), blood lipid levels (low density lipoprotein (LDL), triglycerides, cholesterol), body composition (waist circumference and body weight) and blood pressure (systolic, diastolic).

## Results

In total, 46,500 studies were identified by searching the bibliographic databases PubMed, Scopus, Cochrane Library, EBSCO and IndMED with search strings of keywords. Of these studies, 45270 studies were excluded because they were either irrelevant topics or inappropriate studies. Finally, 1230 articles were screened for further evaluation. A third reviewer monitored the review process to validate the complete study search and selection of most relevant studies for inclusion and monitored the review process. After removing the duplicates and abstract screening, 1216 articles were excluded, because the articles were either reviews, letters to the editor or case studies. This exclusion step resulted in 14 studies after careful manual screening. The reference lists of the existing narrative reviews and meta-analysis were checked and revealed no further relevant missed studies. Finally, 14 studies were eligible for inclusion in our systematic review. Also, some studies failed to mention the association between yoga and metabolic syndrome. Therefore, a final total of 14 studies were included in the systematic review. The study selection is depicted in Fig 1.

Of the 14 studies, six were from India, four from USA, two from China and one each from Sweden and Hong Kong. The total population included in this study was 834 participants from 14 studies. The gender details were available in 10 of the included 14 studies in the systematic analysis. In the total 14 studies, 285 males and 413 females were reported. Our quantitative synthesis demonstrated that 12 studies were randomised control trial, and two were non-randomized control trial. The follow-up period ranged from 4 to 52 weeks. The interventions were yoga practitioners, pre-diabetic, diabetic, obese and metabolic syndrome, as indicated in Table 1.

**Table 1:** Characteristics of the included studies.

S.No	Author and Year	Country	Participation time	Yoga Asanas	Intervention	No of patients	Age and Sex	Control group	No of patients	Age and Sex	Follow-up period	Outcome measures	Study design	Mean and SD values
1	Corey SM et al. 2014 [81]	USA	NA	Restorative yoga intervention	Yoga participants	88	21–65 yrs	Stretching participants	83	21–65 yrs	One year	Salivary cortisol and Psychosocial measurements	Two-arm RCT	NA
2	Hegde et al. 2013 [49]	India	2007–2008	18 Yoga asanas	Pre-diabetics patients	14	30 and 75 yrs: Male-6: Female-8	Computer generator randomised list	15	30 and 75 yrs Male-8: Female-7	3 months	Oxidative stress, glycemic status, Blood pressure, Anthropometry	RCT	Provided
3	Kanaya Am et al 2014 [72]	USA	2009–2012	Restorative yoga intervention	Patients	88	21–65 yrs: F-65: M-23	Stretching participants	83	21–65 yrs F-59: M-24	48 weeks	fasting and 2-hour glucose, HbA1c, triglycerides, HDL Cholesterol	RCT	Provided
4	Keerthi GS et al. 2017 [66]	India	September 2013 to April 2016.	Asanas and pranayama, Meditation and Relaxation	Pre-diabetics and Diabetics	124	18–45 yrs Prediabetics- M-69/F-55: Diabetics-M-64/F-60	Healthy controls	62	M-34/F-28	12 weeks	Biochemical measures, Anthropometric measures, Bp, plasma glucose	RCT	Provided
5	Lau Caren et al. 2015 [83]	China	May 2010 -Jan 2011	57 Yoga Poses	Yoga Practitioners	87	18 and Above M-34: F-53	Non-Yoga practitioners	88	18 and Above M-30: F-56	12 weeks	Metabolic risk factors, Mets z score, Glucose, BP, triglyceride, Smoking, Body weight	Non-RCT	Provided
6	McDermott KA et al. 2014 [69]	India	Oct-Nov 2004	8 types of Yoga asanas and chanting	Yoga Practitioners	21	Age -NA Male-9 Female-12	Non-yoga practitioners, only physical exercises	20	Age -NA; Male-7 Female-13	8 weeks	Diabetic Risk Factors and Physiological Risk Factors	RCT	Provided
7	Netam et al. 2015 [67]	India	Dec 2011-Dec 2012	Asanas and pranayama	overweight/obese individuals	34	M-21: F-13	Pre-baseline	34	M-21: F-13	one month	IL-6, 25-OH-vitamin D and diabetes risk factors	Non-RCT	Provided
8	Siu et al. 2015 [84]	Hongkong	Nov 2010 and Aug 2013	34 Yoga asanas	Metabolic syndrome	84	30–80 M-23: F-75	Computer generator randomised list	98	30–80 M-24: F-60	One year	Wait, BP, glucose, triglycerides, HDL-C, Heart rate	RCT	Provided
9	Sohl et al 2016 [71]	USA	June 2013 to January 2014	12 Yoga-asanas	Metabolic syndrome patients	33	M-17: F-16	education only	33	M-16; F-17	12 weeks	Biometric and Patient-reported constructs	RCT	Provided
10	Supriya et al. 2017 [73]	China	NA	18 Yoga-asanas	Metabolic syndrome patients associated with Diabetes & Cardiovascular diseases	52	M-17: F-35	Computer generator randomised list	45	M-17: F-28	One year	Blood glucose, triglycerides, HDL-C, Waist circumference	RCT	Provided
11	Tyagi A et al. 2014 [68]	India	Nov and Jan 2012	Yoga teachers, Yoga therapists and Yoga active person	Yoga Practitioners	NA	18 yrs to 55 yrs	Non-yoga practitioners	NA	18 yrs to 55 yrs	6 months	Anthropometric measures, BP measurement, metabolic measures	RCT	NA
12	Wolff M et al. 2013 [85]	Sweden	1 May 2008 and 31 January 2010	NA	three groups: through Yoga practitioner, Yoga at home, a control group who take mediation from a general practitioner	56	20–80 yrs	Computer generator randomised list	27	20–80 yrs	12 weeks	Bp, glucose, HBA1c, Cholesterol	three-arm RCT	Provided
13	Yadhav et al. 2018 [70]	India	September 2013 to April 2016.	Asanas and pranayama, Meditation and Relaxation	Metabolic syndrome patients	130	20–45 years Gender number details not provided	dietary intervention	130	20–45 years Gender number details NA	12 weeks	Weight, Height, Body mass, BP, Glucose, Insulin, HDL-C, Insulin, Triglyceride	RCT	Provided
14	Yang K et al. 2011 [86]	USA	NA	Asanas and pranayama	Patients	23	45 and 65 years, M-2; F-21	Pre-baseline	23	45 and 65 years, M-2; F-21	3 months	Weight, Bp, Glucose, Insulin, Lipid panel	Randomised Control Trial	Provided

### Analysis of the overall effects of yoga intervention

Fourteen studies reported FBG as a primary outcome. The effects of yoga compared to control conditions were observed for the pooled SMD (glycemic control) of FBG was -0.064 mg/dL (95% CI -0.201 to 0.074); PPBG was 0.268 mg/dL (95% CI 0.006–0.530); and HbA1c was 0.021% (95% CI -0.164 to 0.205). Relative to controls, the subgroup analysis of other measures of T2DM management revealed effects of yoga compared to controls for lipid profile, body compositions and blood pressure. The efficacy of yoga compared to control for pooled SMD (lipid profile) of LDL was -0.090 mg/dL (95% CI -0.270 to 0.090); triglycerides was -0.148 mg/dL (95% CI -0.285 to -0.012); and total cholesterol was -0.058 mg/dL (95% CI -0.220 to 0.104). The pooled SMD (body composition) for waist circumference was 0.023 cm (95% CI -0.206 to 0.251) and body weight was 0.045 kg (95% CI -0.338 to 0.427). The mean SMD (blood pressure) for systolic blood pressure was -0.058 mm Hg (95% CI -0.168 to 0.053); and diastolic blood pressure was 0.010 mm Hg (95% CI -0.098 to 0.117).

### Sensitivity analysis

In the included studies with a low risk of selection bias using random sequence generation, all studies were located. A significant effect of yoga in comparison to control group conditions was observed in pooled SMD of the primary outcome measure. In the case of allocation concealment, which is a source of bias, most of the studies except six generated high and unclear Risk of Bias assessment (ROB). This could imply that the results were not distinguishable from potential bias. In RCTs with low risk of detection bias (Blinding of Outcome Assessment), thirteen studies were located. In RCTs with a low risk of attrition bias (Incomplete Out-come Data), one study was identified. The risk of biases is shown in Table 2.

**Table 2:** Risk of bias assessment of the included studies.

S.No	Author and Year	Random sequence generation (Selected bias)	Allocation concealment (selection bias)	Blinding of participants and personnel (performance bias)	Blinding of outcome assessment (detection bias)	Incomplete outcome data (Attrition bias)	Selective reporting (reporting bias)	Other bias	Jadad Score
1	Corey SM et al. 2014 [81]	Low risk	unclear	High risk	High risk	High risk	Low risk	Unclear	**2**
2	Hegde et al. 2013 [49]	Low risk	Low risk	High risk	Low risk	Low risk	Low risk	Low risk	**4**
3	Kanaya Am et al. 2014 [72]	Low risk	Low risk	High risk	Low risk	Low risk	Low risk	Low risk	**3**
4	Keerthi GS et al. 2017 [66]	Low risk	High risk	Low risk	Low risk	Low risk	Low risk	Low risk	**3**
5	Lau Caren et al. 2015 [83]	Low risk	Low risk	Low risk	Low risk	Low risk	Low risk	Low risk	**3**
6	McDermott KA et al. 2014 [69]	Low risk	Low risk	Low risk	Low risk	Low risk	Low risk	Low risk	**4**
7	Netam et al. 2015 [67]	Low risk	High risk	Low risk	Low risk	Low risk	Low risk	Low risk	**3**
8	Siu et al. 2015 [84]	Low risk	High risk	Low risk	Low risk	Low risk	Low risk	Low risk	**3**
9	Sohl et al. 2016 [71]	Low risk	High risk	Low risk	Low risk	Low risk	Low risk	Low risk	**3**
10	Supriya et al. 2017 [73]	Low risk	High risk	Low risk	Low risk	Low risk	Low risk	Low risk	**3**
11	Tyagi A et al. 2014 [68]	Low risk	Unclear	Low risk	Low risk	Low risk	High risk	Unclear	**2**
12	Wolff M et al. 2013 [85]	Low risk	High risk	Low risk	Low risk	Low risk	Low risk	Low risk	**3**
13	Yadhav et al. 2018 [70]	Low risk	Low risk	Low risk	Low risk	Low risk	Low risk	Low risk	**4**
14	Yang K et al. 2011 [86]	Low risk	Low risk	Low risk	Low risk	Low risk	Low risk	Low risk	**4**

Jadad Score:

Randomisation (0–2 points), blinding (0–2 points)

Dropouts and withdrawals (0–1 point)

Score ≤2 indicates low quality, whereas a score of ≥3 indicates high quality

### Primary outcome -Glycemic control

The beneficial outcomes were noticed in fasting blood glucose, but not in postprandial glucose and glycosylated hemoglobin (HbA1c).

#### Fasting blood glucose

The meta-analysis revealed that yoga is beneficial in the control of blood sugar levels compared to control group conditions.

#### Does yoga intervention decrease fasting blood glucose level?

**Fig 2:**
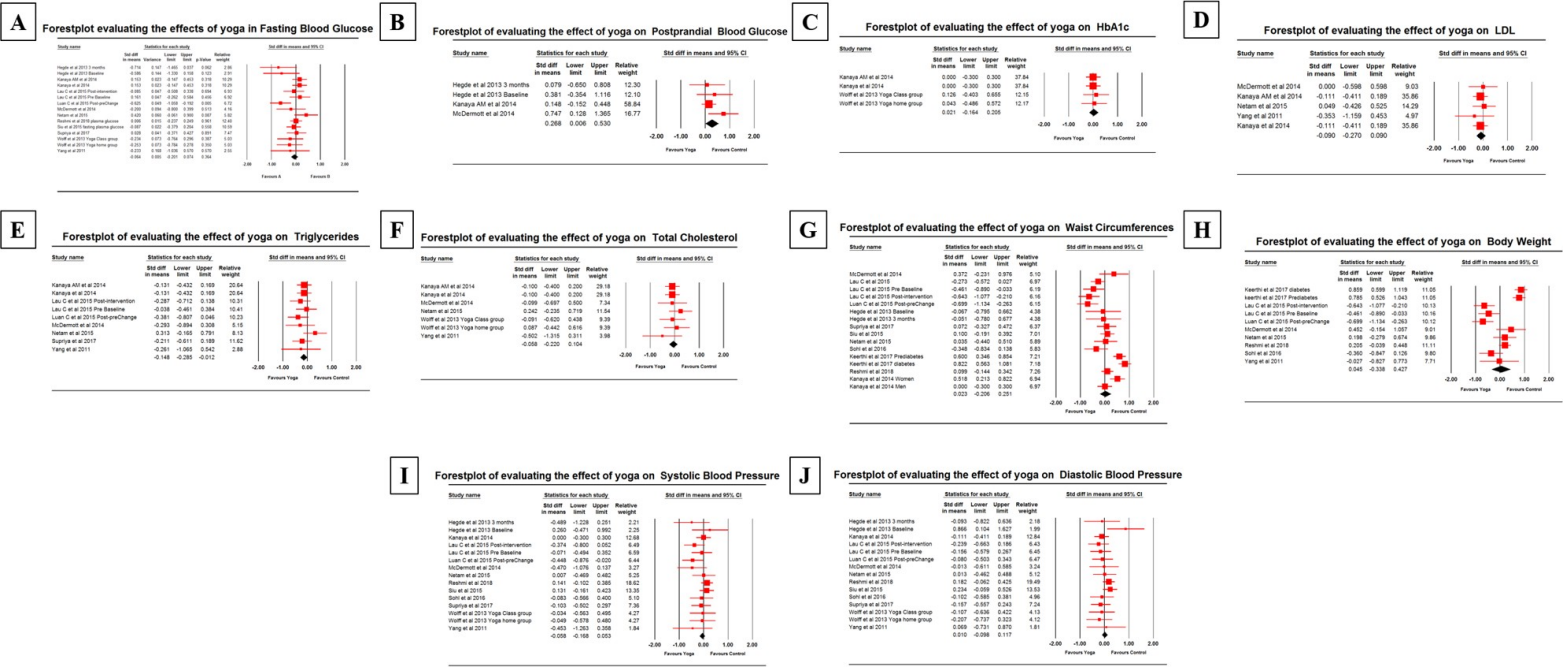
Forest plot of the effect of yoga on glycemic control, lipid profile, body composition and blood pressures in prediabetes. A) fasting blood glucose, B) Postprandial blood glucose, C) Glycosylated hemoglobin (HbA1c), D) LDL, E) Triglycerides, F) Total Cholesterol (TC), G) Waist circumferences, H) Body weight, I) Systolic blood pressure (SBP) and J) Diastolic blood pressure (DBP). The pooled SMD data were calculated and analysed using CMA software (version 3.3.070, USA). The black diamond represents the pooled effect estimate of SMD of the included. The red square with line indicates the effect size of miRNA of the included studies with 95% confidence interval.

Fourteen studies provided the SMD values for the meta-analysis on the fasting blood glucose; the point estimate of effect size for random effects of SMD was -0.064 (95% CI -0.201 to 0.074) (Fig 2).

#### How much does the effect size of yoga efficacy differ across studies?

The Q statistics provide a test of the null hypothesis that all RCTs in the meta-analysis share a standard effect size. If all RCTs shared the same effect size, the expected value of Q would be equal to degrees of freedom. The Q value is 21.75 with 14 degrees of freedom and a corresponding *P-value* = 0.084. The I^2^ statistic indicates the proportions of the observed variance that reflects the difference in actual effect sizes rather than sampling error. Here I^2^ was 35%. T^2^ (tau^2^ is the variance of true effect sizes and tau^2^ = 0.024.

#### Postprandial blood glucose

Four studies provided the SMD values for the meta-analysis on the postprandial blood glucose; the point estimate for random effects ratio of SMD was 0.268 (95% 0.006 to 0.530) with the degrees of freedom (df) = 3, I^2^<7.756, *P-value* = 0.354 and tau^2^ = 0.007.

#### Glycosylated hemoglobin (HbA1c)

Four studies provided the SMD values for the meta-analysis on the HbA1c; the point estimate for random effects ratio of SMD was 0.021 (95% CI-0.164 to 0.205) with the degrees of freedom (df) = 3, I^2^<0.000, *P-value* = 0.978 and tau^2^ = 0.000.

### Secondary outcomes

#### Lipid profile

Yoga intervention was successful in reducing all three measures of the lipid profile of yoga participants who exhibited lower LDL, triglycerides and total cholesterol compared to controls.

#### Low-density lipoprotein (LDL)

Five studies provided the SMD values for the meta-analysis on the low-density lipoprotein; the point estimate for random effects ratio of SMD was -0.090 (95% CI -0.270 to 0.090) with the degrees of freedom (df) = 4, I^2^<0.000, *P-value* = 0.930 and tau^2^ = 0.000.

#### Triglycerides

Nine studies provided the SMD values for the meta-analysis on the triglycerides; the point estimate for random effects ratio of SMD was -0.148 (95% CI -0.285 to -0.012) with the degrees of freedom (df) = 8, I^2^<0.000, *P-value* = 0.669 and tau^2^ = 0.000.

#### Total cholesterol

Seven studies provided the SMD values for the meta-analysis on the total cholesterol; the point estimate for fixed effects ratio of random effects ratio of SMD was -0.058 (95% CI -0.220 to 0.104) with the degrees of freedom (df) = 6, I^2^<0.000, *P-value* = 0.792 and tau^2^ = 0.000.

#### Body composition

On subgroup analysis, no effect on body composition was observed in the yoga intervention group as compared with the control conditions.

#### Waist circumference

Yoga intervention was less successful in decreasing waist circumference. Sixteen studies provided the SMD values for the meta-analysis on the waist circumference; the point estimate for random effects ratio of SMD was 0.023 (95% CI -0.206 to 0.251) with the degrees of freedom (df) = 15, I^2^<83.872, *P-value* = 0.000 and tau^2^ = 0.171.

#### Body weight

Ten studies provided the SMD values for the meta-analysis on the body weight; the point estimate for random effects of SMD 0.045 (95% CI -0.338 to 0.427) with the degrees of freedom (df) = 9, I^2^<89.974, *P-value* = 0.000 and tau^2^ = 0.328.

#### Blood pressure

The aggregated results suggested that yoga intervention was successful in reducing systolic blood pressure, but not diastolic, blood pressure.

#### Systolic blood pressure (mmHg)

Participants in yoga intervention showed a decrease in systolic blood pressure compared to controls. Fifteen studies provided the SMD values for the meta-analysis on the systolic blood pressure; the point estimate for random effects ratio of SMD-0.058 (95% CI -0.168 to 0.053) with the degrees of freedom (df) = 14, I^2^<3.352, *P-value* = 0.414 and tau^2^ = 0.002.

#### Diastolic blood pressure (mmHg)

Fourteen studies provided the SMD values for the meta-analysis on the diastolic blood pressure; the point estimate for random effects ratio of 0.010 (95% CI -0.098 to 0.117) with the degrees of freedom (df) = 14, I^2^<0.000, *P-value* = 0.485 and tau^2^ = 0.000. The effect size estimate of Glycemic control, lipid profile, body composition and blood pressure is shown in Table 3.

**Table 3:** Effect size estimate of outcome measures in prediabetes state (glycemic control, lipid profile, body composition and blood pressure.

Outcomes	Measures	No of studies	Sample size		ES	95% CI		I^2^	Tau^2^	Q	Z
			Yoga	Control		Low	High				
Glycemic control	FBG	15	722	720	-0.037	-0.14	0.067	35.654	0.024	21.757	-0.908
	HbA1c	4	232	220	0.021	-0.164	0.205	0	0	0.196	0.218
	PBG	4	137	135	0.268	0.006	0.53	7.756	0.007	3.252	2.008
Lipid profile	LDL	5	243	234	-0.9	-0.27	0.09	0	0	0.863	-0.984
	Triglycerides	9	424	408	-0.148	-0.285	-0.012	0	0	5.801	-2.133
	Total cholesterol	7	299	288	-0.058	-0.22	0.104	0	0	3.136	-0.701
Body composition	WC	16	1022	1021	0.023	-0.206	0.251	83.872	0.171	93.005	0.194
	Body weight	10	607	608	0.045	-0.338	0.427	89.974	0.328	89.77	0.228
Blood pressure	SBP	15	667	670	-0.058	-0.168	0.053	3.352	0.002	14.486	-1.021
	DBP	15	667	670	0.01	-0.098	0.117	0	0	13.529	0.173

FBG: Fasting Blood Glucose; ES: Effect Size; CI: Confidence Interval; PBG: Prandial Blood Glucose; LDL: Low Density Lipoprotein; WC: Waist Circumference; SBP: Systolic Blood Pressure; DBP: Diastolic Blood Pressure.

### Publication bias

**Fig 3:**
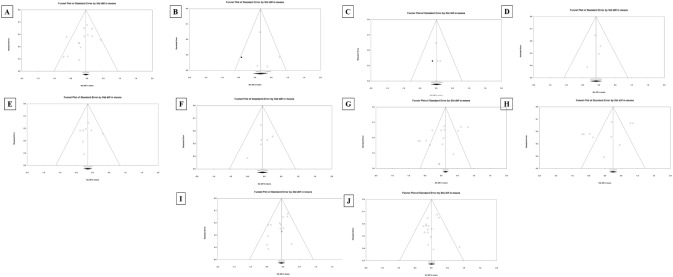
Funnel plot of yoga intervention efficacy on glycemic control, lipid profile, body composition and blood pressure in prediabetes. A) Fasting blood glucose (FBS), B) Postprandial blood glucose, C) Glycosylated hemoglobin (HbA1c), D) LDL, E) Triglycerides, F) Total Cholesterol (TC), G) Waist circumferences, H) Body weight, I) Systolic blood pressure (SBP), and J) Diastolic blood pressure (DBP).

Funnel Plots for all outcome measures are represented in Fig 3. Because the intervention effect estimated from a biased collection of studies would tend to overestimate the exact intervention effect, researches must assess the likely extent of the bias and the impact of this goes on the conclusions. The detailed explanation of the publication bias among the included studies that examined fasting blood glucose is shown in Fig 2 and Table 4 shows the publication bias of remaining outcome measures.

**Table 4:** Publication bias of the included studies.

Key variables	Outcome measures	Classic fail-safe N		Orwin fail-safe N	Begg and Mazumdar			Egger's regression	Duval and Tweedie
		Z value	P value	SDM	Tau	Z value	P value		
Glycemic control	FBG	1.419	0.155	0.132	-0.291	1.575	0.115	-3.65	0.132
	HbA1c	0.313	0.754	0.02	0.8	1.358	0.174	0.723	0.005
	PPBG	-0.942	0.346	-0.09	0	0	1	-0.07	-0.09
Lipid profile	LDL	-0.942	0.346	-0.09	0	0	1	-0.07	-0.09
	Triglycerides	-0.703	0.481	-0.058	-0.15	0.45	0.652	-0.143	-0.058
	Total cholesterol	-2.103	0.035	-0.148	-0.114	0.417	0.676	-0.219	-0.148
Body composition	WC	-1.445	0.148	-0.036	-0.394	2.028	0.042	-1.814	-0.036
	Body weight	2.108	0.035	0.248	-0.088	0.357	0.72	-5.014	0.248
Blood pressure	SBP	-1.656	0.097	-0.053	-0.285	1.484	0.137	-1.749	-0.053
	DBP	-0.02	0.983	0.009	0.171	0.89	0.373	-0.439	0.009

FBG: Fasting Blood Glucose; ES: Effect Size; CI: Confidence Interval; PPBG: Postprandial Blood Glucose; LDL: Low-Density Lipoprotein; WC: Waist Circumference; SBP: Systolic Blood Pressure; DBP: Diastolic Blood Pressure.

#### Classic fail-safe N

This meta-analysis incorporates data from 15 studies, which yield a z-value of -1.44545 and a corresponding 2-tailed *p*-value of 0.14833. Although the pooled result is not statistically significant, the Fail-Safe N -which addresses the concern that the observed significance may be spurious is not relevant.

#### Orwin fail-safe N

First, the mean STD difference in means in the added studies can be a value other than the nil value 0. Second, the criterion value is an effect size rather than a p-value. That is, the Orwin fail-safe N is the number of added studies that will shift the combined STD difference in means past a specified threshold from the starting point of 0.

#### Begg and Mazumdar rank correlation test

The Kendall's tau b is -0.39423, with a 1-tailed p-value of 0.02123 or a 2-tailed p-value of 0.04246.

#### Egger's test of the intercept

The Eggers bias indicator test predicted that the intercept (B0) is -1.81427, 95% confidence interval (-3.67106, 0.04253), with t = 2.11089, df = 13. The 1-tailed p-value (recommended) is 0.02736, and the 2-tailed p-value is 0.05472.

#### Duval and Tweedie's Trim and Fill

The test is in search of missing studies dependent on a fixed effect model and represents missing studies only to the left side of the mean intervention effect. Using these parameters, the method suggests that no studies are missing. Under the fixed effect model, the point estimate and 95% confidence interval for the combined studies is 0.13277 (0.04490, 0.22064). Using Trim and Fill the imputed point estimate is the same value above. Under the random effects model, the point estimate and 95% confidence interval for the combined studies is 0.02255(-0.20574, 0.25084). Using Trim and Fill the imputed point estimate is the same value as mentioned above.

## Discussion

### Summary of the key finding

We conducted as a systematic and meta-analysis to explore the association between yoga intervention and prediabetes state. This study was designed to address whether yoga delays or prevent the progression of diabetes in prediabetic population especially compared to exercise. The meta-analysis examined whether yoga improves glycemic control, lipid profile, body composition and blood pressure in prediabetes individuals. Fasting blood glucose, LDL, triglycerides, total cholesterol and systolic blood pressure were reduced by 0.064 mm Hg, 0.090 mg/dL, 0.148 mg/dL, 0.058 mg/dL, and 0.058 mmHg respectively.

### Consistent with previous systematic review and meta-analysis

To our knowledge, no previous systematic review and meta-analysis have investigated the effect of yoga intervention on the prediabetic state. Therefore, our findings in the prediabetic population are consistent with studies that reported the effects of yoga practice on T2DM. Results on fasting plasma glucose (FPG) levels and lipid profiles are similar to studies that report a general effect of Yoga in diabetic patients [38, 63]. The findings of this systematic review on yoga on prediabetes included 12 RCTs and two non-RCTs and show that a yoga intervention could improve fasting blood glucose, lipid profiles and systolic blood pressure. More specifically, yoga intervention lowered lipid profiles such as LDL, TC, and triglycerides. Similarly, a previously published qualitative review on yoga for T2DM that included 4 RCTs and 21 non-RCTs concluded that yoga might improve glucose tolerance, lipid profiles (LDL, TC), anthropometric measures, and blood pressure in T2DM [64].

Consistent with the previous meta-analysis, Cui and colleagues demonstrated, in their meta-analysis with 12 RCTs with a total of 864 patients, that yoga could significantly decrease FBG, PPBG, HbA1c, TC and LDL levels [11]. Eun and Doi showed in a meta-analysis with 11 RCTs with a total of 993 participants that yoga intervention improves glycemic control and lipid profiles in T2DM [63]. Similar findings were observed in Thind and colleagues’ meta-analysis study on 23 studies investigating glycemic control, lipid profile, blood pressure and body mass index [44]. Kumar and colleagues showed similar observations with 17 studies focusing on three parameters of glycemic control [43]. Jayawardena and colleagues demonstrated that significant reduction in FBG, PPBG, HbA1c and BMI alter in 'Yoga group compared to the control group; however, they did not observe any significant difference between the two groups on lipid parameters, other body composition measures (WC and WHR) and Blood Pressure [65].

### Applicability of evidence

The included studies in the meta-analysis were obtained from the currently available pool of research, in this field and included 12 RCTs and two non-RCTs. There were four studies from the USA, two studies from China, 1 study from Hongkong, six studies from India and 1 study from Sweden. The studies were drawn using the defined inclusion and exclusion criteria. This meta-analysis included studies with diverse age and ranged from 18 to 75 years old. The studies included 285 men and 413 women. The conclusive findings apply to diverse population and age groups.

Approximately 50% of studies [49, 66–70] and the first author from three studies that were conducted in other countries [71–73], were of Indian origin. Because yoga is centered part of Indian tradition and culture, the intervention would be more effective in this nation. Indeed, the implementation of yoga might be different India. For example, Kumar and colleagues revealed that Indian experimental studies had offered yoga intervention for six days a week in the majority of cases whereas studies performed outside countries delivered their intervention on two days per week [43]. Cramer and colleagues highlighted that the key reasons for the positive clinical outcomes and performance in Indian yoga studies could be that yoga interventions are more intense compared to non-Indian trials. They also added that the key reason for better performance and positive outcomes in Indian yoga studies could be the difference in skills of the yoga trainer trained and practising in India [74]. The findings from this systematic review and meta-analysis could apply to the clear majority of healthy as well as to non-diabetic people with a high risk for T2DM in clinical practice.

### Quality of evidence

In this meta-analysis and subgroup analysis, we determined whether the outcome measures FBG, PPBG, HbA1c, TC, Triglycerides, LDL, waist circumferences, Body weight, SBP, and DBP from different studies exhibited clinical and statistical heterogeneity across the studies. Therefore, the estimated effect size of the standard mean difference between yoga interventions and control conditions for prediabetes state were calculated by using random-effects models in our analysis.

The quality assessment of these included studies was performed using the Jadad score and investigated key rigorous study parameters such as randomisation, blinding, allocation concealment, usage of multiple interventions and adjustment for confounders, dropouts and withdrawals. The included studies exhibited diverse parameters in this systematic review, indicating a moderate to high-quality level score [63, 75, 76]. Publication bias for ten different outcomes of T2DM management in prediabetes state was investigated using six different tools including inverted funnel plot, Orwin fail-safe N test, Classic fail-safe N test, Begg and Mazumdar Rank Correlation test, Harbords-Eggers test of intercept and Duval and Tweedie’s Trim and Fill method. Publication bias can compromise the integrity of any meta-analysis. Without assessing publication bias could be conveyed as null results or missing studies. Our findings showed that the publication bias was unlikely to occur in this meta-analysis. However, in two outcomes measures including body weight and total cholesterol, the number of missing studies would bring p-value >0.05 was 2, and thus these parameters might exhibit publication bias.

### Strengths and limitations

This systematic review and meta-analysis are the first to explore the impact of a yoga intervention for the prediabetes state. This systematic review and meta-analysis were performed by Preferred Reporting Items for Systematic Reviews and Meta-Analyses (PRISMA) guidelines [40], and the protocol has been registered in the International prospective register of systematic reviews (PROSPERO). The included studies comprised of 12 RCTs (one three-arm RCT, two arm RCTs and 9 RCTs) and two non-RCTs. These studies investigated a diverse population with a wide range of age groups. In this systematic review and meta-analysis, clinical applicability and quality of evidence of the included studies were compared with ten different outcome measures between yoga and control conditions.

The interpretation of the effect size of glycemic control and other three key outcomes is limited by the non-availability and insufficient reporting of PPBG, HbA1c and other outcome measures. These issues might influence the inadequate definition of subgroups, substantial differences in interventions, control conditions, and outcome assessment [77, 78].

The follow-up duration in the RCT in most of the included studies ranged from 4 weeks to 1 year. The authors reported limited information about RCT study participant’s withdrawals and lost to follow. The outcome measures of the included studies were primarily calculated after follow-up. No reporting of intervention fidelity or inadequate reporting of baseline measures and demographic characteristics of participants (sample size) were observed. Two RCTs have reported three phases of yoga: Asanas, Pranayama and meditation. However, other information on yoga such as yoga postures, style of yoga (i.e. Vinyasa, Ashtanga, Iyengar, Kripalu, and Kundalini), intensity and duration of individual yoga asana or posture, type and duration of mediation were not reported in detail [44].

### Future recommendations

Future studies must follow CONSORT statement for RCTs and TREND statement for non-RCTs to avoid inadequate reporting of sample size, randomization, allocation concealment, intention-to-treat analysis, and blinding of at least outcome assessors [44, 79, 80]. Thind and colleagues proposed that future studies should also report and record any adverse effects or injuries associated with yoga intervention [44].

Only a single study investigated the effects of yoga on oxidative stress [49], cortisol [81], inflammatory marker [67], or biometric measures [71], with only two studies available on anthropometric measures [66, 68]. All these parameters have been demonstrated to be important factors for cardiovascular (what?) and T2DM [74]. Therefore, future studies should consider evaluating these measures in their assessment. Future research focuses on yoga interventions with an emphasis on asanas, pranayama and meditation in addition to sathivka (healthy vegetarian diet) diet and yogic life-style [74, 82].

## Conclusion

This meta-analysis of yoga for diabetes and associated factors revealed evidence for the potential clinically important effect of yoga on ameliorating diabetes factors such as fasting blood glucose, postprandial blood glucose, body weight, systolic blood, diastolic blood pressure, LDL, triglycerides, total cholesterol and HbA1c. However, well-designed RCTs are needed to examine the long-term efficacy of yoga intervention not only for the risk of T2DM but also metabolic syndrome and cardiovascular disease.

## Supporting information

S1 Table Diabetes—PRISMA checklist. (DOC)

## Abbreviations

CI: Confidence Interval
CMA: Comprehensive Meta-Analysis
DBP: Diastolic Blood Pressure
df: degrees of freedom
FBG: Fasting Blood Glucose
HbA1c: Hemoglobin A1c
HDL: High Density Lipoprotein
LDL: Low-Density Lipoprotein
PICOS: Participants, Intervention, Control, Outcomes
PPBS: Post-Prandial Blood Glucose
PRISMA: Preferred Reporting Items for Systematic Review and Meta-Analysis
PROSPERO: International prospective register of systematic reviews
RCTs: Randomized Controlled Trials
SBP: Systolic Blood Pressure
SMD: Standardized Mean Difference
T2DM: Type 2 diabetes mellitus
